# A brief history of bird flu

**DOI:** 10.1098/rstb.2018.0257

**Published:** 2019-05-06

**Authors:** Samantha J. Lycett, Florian Duchatel, Paul Digard

**Affiliations:** The Roslin Institute, University of Edinburgh, Edinburgh, UK

**Keywords:** avian influenza virus, epidemiology, phylogenetics, pandemic, zoonotic

## Abstract

In 1918, a strain of influenza A virus caused a human pandemic resulting in the deaths of 50 million people. A century later, with the advent of sequencing technology and corresponding phylogenetic methods, we know much more about the origins, evolution and epidemiology of influenza epidemics. Here we review the history of avian influenza viruses through the lens of their genetic makeup: from their relationship to human pandemic viruses, starting with the 1918 H1N1 strain, through to the highly pathogenic epidemics in birds and zoonoses up to 2018. We describe the genesis of novel influenza A virus strains by reassortment and evolution in wild and domestic bird populations, as well as the role of wild bird migration in their long-range spread. The emergence of highly pathogenic avian influenza viruses, and the zoonotic incursions of avian H5 and H7 viruses into humans over the last couple of decades are also described. The threat of a new avian influenza virus causing a human pandemic is still present today, although control in domestic avian populations can minimize the risk to human health.

This article is part of the theme issue ‘Modelling infectious disease outbreaks in humans, animals and plants: approaches and important themes’. This issue is linked with the subsequent theme issue ‘Modelling infectious disease outbreaks in humans, animals and plants: epidemic forecasting and control’.

## 1. Introduction

### (a) Influenza viruses

Influenza viruses are part of the *Orthomyxoviridae* family [1] and are negative sense single-stranded RNA viruses with segmented genomes. There are four main influenza virus species: A, B, C and D. Type A viruses are known to infect a wide variety of birds and mammals, while the other species have more restricted host ranges. Influenza A viruses (IAV), including all avian influenza viruses, possess eight separate genomic segments ranging in size between 890 and 2341 nucleotides [1,2]. Like other RNA viruses, influenza viruses have a fast mutation rate, typically accumulating two to eight substitutions per 1000 sites per year [3]. Segmentation further increases the evolutionary speed of the virus by permitting exchange of genes between virus strains that co-infect cells in the same host, a process known as reassortment. The genome segments of IAV encode ten core polypeptides, including: three subunits of a viral polymerase, a nucleoprotein, three transmembrane proteins (haemagglutinin (HA), neuraminidase (NA) and the M2 ion channel), a matrix protein M1 and ‘non-structural’ proteins NS1 and NS2/NEP, as well as a virus strain-dependent suite of non-essential accessory proteins [4]. The HA and NA surface proteins are antigenic, very diverse, encoded on separate segments and split into 18 and 11 subtypes, respectively. Apart from the recently discovered bat-specific H17, H18, N10 and N11 proteins [5,6], all of the subtypes have been found in avian species, whereas only a subset of the others have been detected in mammals. The other six segments are often considered as encoding the ‘internal’ genes. Although there is continuous global circulation of IAV in humans, due to the connectivity of the population [7], the majority of the diversity is in avian species and the reservoir population is avian [2]. Therefore, understanding the general global patterns of IAV epidemiology in birds will help elucidate the origins of past pandemics and could help inform predictions about future events.

### (b) Major IAV lineages

**Figure 1.**
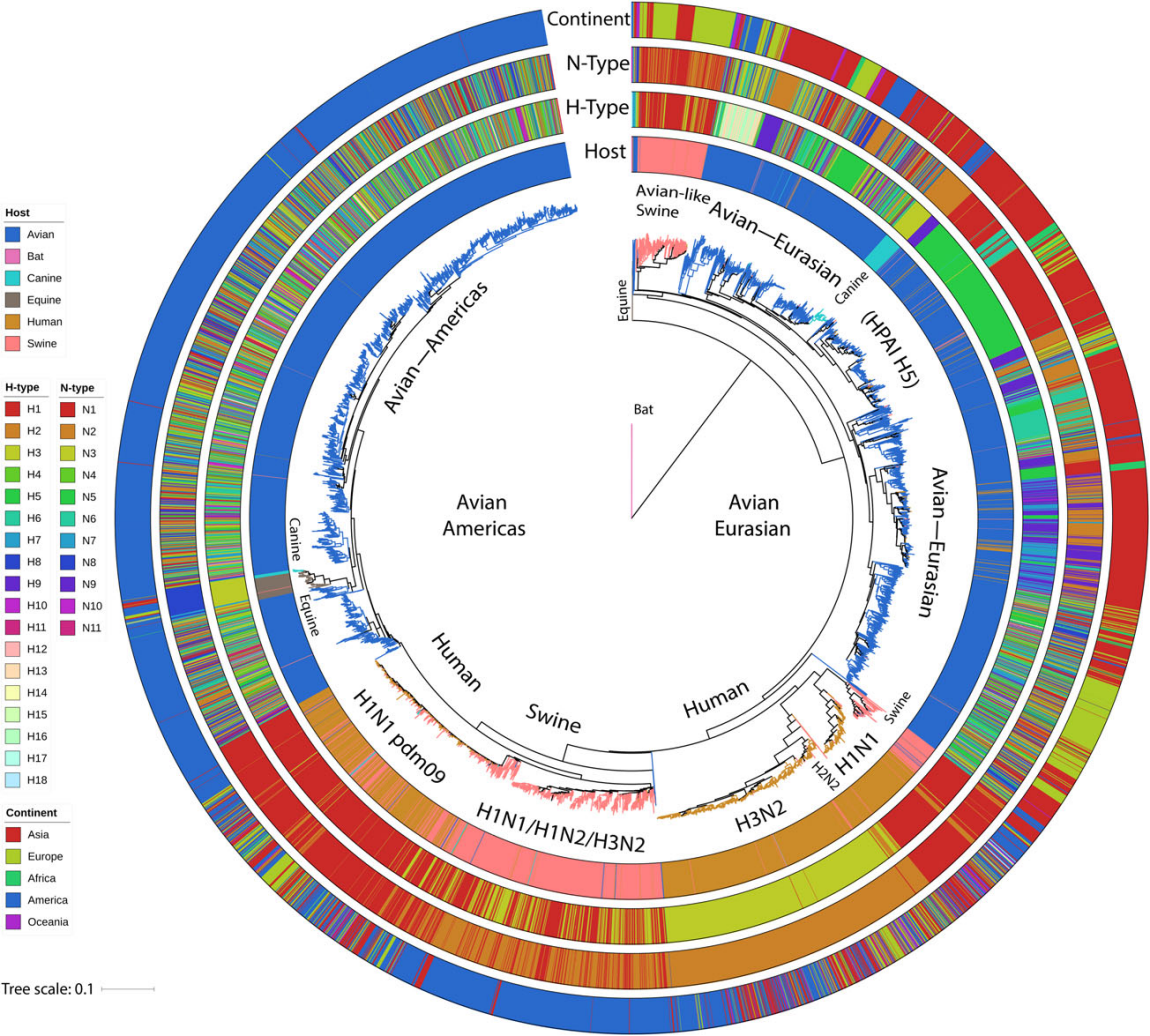
PB2 phylogeny of a stratified sample of all influenza subtypes. Tips in the circular neighbour-joining tree are coloured by host: blue, avian; pink, swine; orange, human; green, canine; brown, equine; purple, bat. The rings from inner to outer are: host-type, haemagglutinin subtype (H-type), neuraminidase subtype (N-type) and continent of isolation. The tree shows (clockwise) bats at the root, the avian—Eurasian lineage with many subtypes, human seasonal H1N1, H2N2 and H3N2 spread through all the continents, H1N1, H1N2, H3N2 swine influenza, the H1N1 pandemic swine influenza lineage in humans and swine, and the avian—Americas lineage with many subtypes.

Figure 1 shows a phylogenetic tree from 8809 nucleotide sequences of segment 1, which encodes the polymerase basic 2 (PB2) subunit of the viral polymerase, with major hosts and subtypes marked. The sequences in the tree are a stratified subsample (one or two per host-type, subtype, country or state and year) of all the virus isolates with complete genome sequences in Genbank, obtained through the Influenza Virus Resource database [8] (approx. 40 000 in July 2018) and represent the known diversity of IAV. Details of the sequences as well as the alignments files and tree files for all internal segments can be found in the electronic supplementary material. Major lineages for avian, swine, human, equine and canine hosts can be observed, although cross-species transmissions are quite common. As indicated in the figure, reassortment of the surface protein-encoding segments is rife in avian virus lineages [9,10] and present to some extent in swine lineages [11,12], but is generally uncommon for the human, equine and canine lineages.

### (c) Fowl plague is avian influenza

Severe non-bacterial outbreaks with high mortality rates in domestic birds have been recorded since the late 1800s (reviewed in [13,14]). In the nineteenth and early twentieth centuries, these outbreaks were termed ‘fowl plague’, and it was not until 1955 that Schafer determined that ‘fowl plague virus’ (FPV) was indeed a type of IAV, with similar internal antigens to human and swine influenza viruses [15]. Sequencing studies performed many years later resulted in the identification of the highly pathogenic avian influenza (HPAI) virus strains responsible for these outbreaks as H7 subtype IAVs, including A/chicken/Brescia/1902 (H7N7) [16], A/FPV/Weybridge/1927 or A/FPV/Dutch/1927 (H7N7) [13,17] and A/chicken/FPV/Rostock/1934 (H7N1) [18]. In 1959, an antigenically different HPAI H5 subtype was found in a chicken farm in Scotland (represented by A/chicken/Scotland/1959 (H5N1) [17]), while in 1961 an H5N3 strain was isolated from a wild common tern *(Sterna hirundo)* in South Africa (A/tern/South Africa/61 (H5N3) [19]). Because of the highly pathogenic phenotype of these first H5 and H7 isolates, it was parsimonious to consider all H5 and H7 viruses to be similarly virulent. However, this was reconsidered after the isolation of low pathogenic avian influenza (LPAI) H5 and H7 strains from ducks in the 1950/1960s and from turkeys in the 1960s/early 1970s (e.g. A/turkey/Ontario/77332/66 (H5N9) [20] and A/turkey/Oregon/71 (H7N3) [21]). Since then, an enormous variety of LPAI and HPAI H5 and H7 subtypes have been isolated from domestic and wild birds, as well as the viruses bearing the majority of all other possible combinations of H1–H16 and N1–N9 surface glycoproteins [2,22–24]. The molecular basis for the strikingly different virulence phenotypes seen with H5 and H7 viruses has also been elucidated (see box 1).

Box 1. **The molecular basis for high and low pathogenic phenotypes of H5 and H7 strains of IAV.**

**Figure 2.**
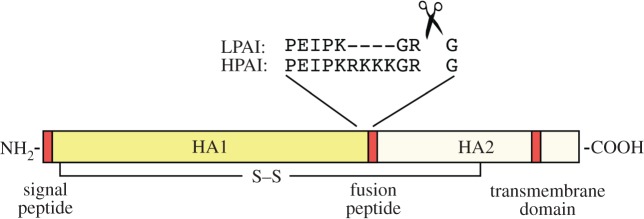
Cartoon depicting post-translational processing of a linear HA monomer. HA1 and HA2 domains are indicated, as is the linking disulfide bridge. Red boxes indicate hydrophobic regions involved in membrane interactions. Example cleavage sequences from low and high pathogenicity H5 viruses are shown.

HA is synthesized on the endoplasmic reticulum as a precursor HA0 polypeptide, assembled into a trimer, glycosylated and transported to the cell surface. At this point, it can be incorporated into budding virus and is active as a receptor binding molecule. However, it is incapable of promoting membrane fusion and thus virus entry into the next cell, until a post-translational cleavage event has taken place to separate the HA1 and HA2 domains and liberate a fusion peptide at the new N-terminus of HA2 (figure 2). In LPAI strains of virus, cleavage is performed extracellularly by host proteases present on mucosal surfaces, after a single basic residue. By contrast, HPAI strains have an expanded multi-basic sequence that allows intracellular processing via ubiquitous furin-like proteases; this has the consequence of expanding the tissue tropism of the virus and facilitating systemic disease [25–29]. This phenomenon is well established for H5 and H7 HAs, but for reasons that are unclear, has not been seen outside of the laboratory for other HA subtypes.

### (d) Avian influenza and human pandemics

One hundred years ago, in 1918, the ‘Spanish flu’ pandemic, caused by an H1N1 influenza virus is estimated to have contributed to the deaths of around 50 million people [30]. Since then, three other human IAV pandemics have occurred: H2N2 in 1957 (Asian flu), H3N2 in 1968 (Hong Kong flu), and H1N1 again in 2009 (swine flu). In each case, IAV strains bearing segments coding for antigenically novel NA and/or HA surface protein(s) rapidly spread through a human population with no or little prior immunity. The relationship between fowl plague, avian influenza and human influenza was not apparent before the 1950s, but by 1967 Pereira, Tumova & Webster suggested that the human H2N2 and H3N2 pandemic viruses might have had an avian origin on the basis of antigenic cross-reactivity [31].

As soon as IAVs were sequenced (e.g. [18]), phylogenetic analyses started to show how avian and human viruses were related, and how this relationship could vary according to the segments involved. Such studies unambiguously confirmed the avian virus origin of the human 1957 and 1969 pandemic glycoprotein genes [32,33]. The complete sequences of 1918 human H1N1 viruses are also available (e.g. A/Brevig Mission/1/18 (H1N1)) despite this pandemic pre-dating the identification of IAV as the causative agent [34], having been obtained direct from tissue samples of victims [35]. However, it has been difficult to infer the host species of the ancestor(s) of the 1918 pandemic virus, since there are only three partial sequences of HA or NP from contemporary avian isolates (obtained from museum samples collected between 1915 and 1919) [36,37], and most of the earliest other avian and swine virus sequences are from samples from the 1930s [38]. Although the human 1918 H1N1 sequences form a group with the contemporaneous classical swine H1N1 lineage, analysis of the polymerase gene sequences and time-scaled phylogenetic studies indicate that these 1918 human IAV segments probably do have an avian origin [39,40].

The subsequent two human pandemics (1957 and 1968) were not caused by completely avian-origin viruses, but were rather reassortant viruses with avian-origin HA, PB1 polymerase and (for the 1957 pandemic) NA segments [33,41–43]. The N2 neuraminidase in the 1968 strain, however, was a continuation of the avian N2 previously introduced in the human population in 1957 [33]. The 2009 H1N1 ‘swine flu’ pandemic was a result of reassortment between different strains of IAV that had been circulating in swine for at least 10 years [44], but these precursor swine strains all had segments tracing back to avian origins some 30 years previously [44,45].

Sporadic infections of humans with a limited number of avian virus subtypes (H5, H6, H7, H9, H10) have also been known to occur directly from avian sources, but without as yet leading to sustained human to human transmission [46–52]. Typically, these infections are severe in humans, often causing death, and potential zoonotic epidemics are of ongoing concern. Specific episodes with H5 and H7 viruses are considered in more detail later.

## 2. Global patterns of avian influenza circulation

Water fowl, especially Anseriformes (ducks, geese and swans) and Charadriiformes (gulls, terns and sandpipers), are thought to be the natural reservoir of IAV [2,53], and infection in these host species is not only typically low pathogenic but can be asymptomatic [2,54–57]. It has also been shown that migratory birds may carry HPAI as well as LPAI viruses asymptomatically over long distances [53,58–60], and that avian IAV lineages can spread along migratory flyways [61–66]. For example, remote sensing and phylogenetic analyses showed that the distribution of H5N1 viruses in Eastern Asia followed wild bird migratory flyways in the time period 2003–2012 [63].

Transmission between places and host species can be inferred by phylodynamic and phylogeographical analyses [67,68], and these techniques are particularly suitable for understanding avian influenza systems since they make use of the fast-evolving viral sequence data to reveal dispersion patterns (see box 2 for details). Phylogeographic analyses have revealed the role of migratory wild birds in the intra-continental circulation of LPAI in North America [61,62,89], and have implicated wild birds following North American flyways in the introduction of H7N3 strains into Mexico in 2012–2013 [64]. Similarly phylogeographic techniques have also been used to show the effects of HPAI H5N1 transportation by different bird species across Asia [90] and that the spread of LPAI H9N2 strains in Asia was a combination of long-range distribution by wild birds coupled with more localized spread via the domestic bird trade [91].

Box 2. **Inference of transmission routes using phylodynamics and phylogeography.**

**Figure 3.**
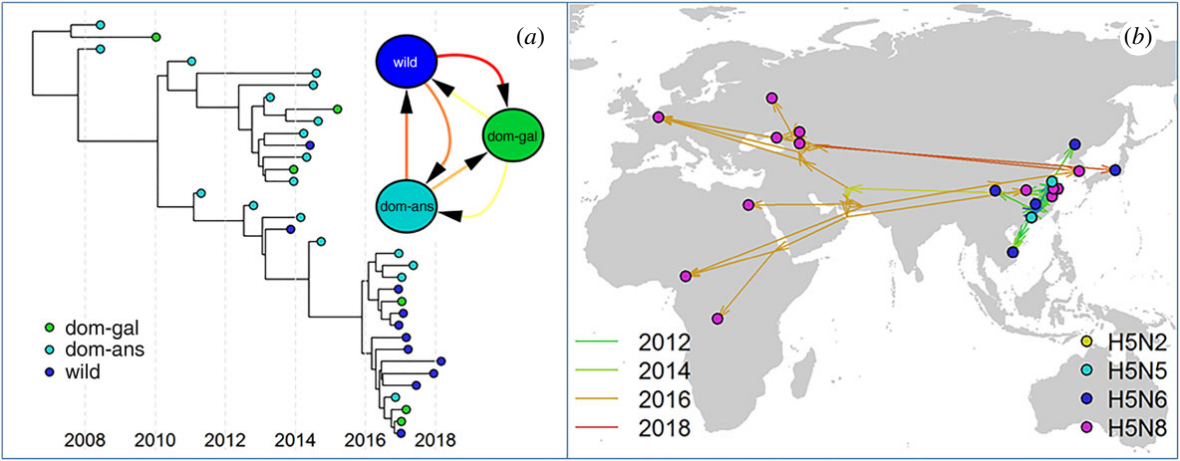
Example of a time-scaled phylogenetic tree with tips coloured by host-type and the discrete trait host model (*a*); and the same tree mapped into space with continuous spatial coordinates with tips coloured by subtype (*b*).

Viral sequence data sampled over a period of time, spatial locations and different host species can be used to infer transmission patterns (e.g. [63,64,67–71], figure 3). Typically for IAV, time-scaled phylogeny reconstruction is often performed using the programme BEAST (Bayesian Evolutionary Analysis Sampling Trees) [72,73] in which trees and relaxed molecular clock models used to represent the relationship between genetic distance and time, and other parameters, are jointly inferred.

To infer transmission rates between discrete locations or hosts, or to model subtype changes (for example, the change of NA subtype with respect to a tree made from HA sequences), phylogenetic analysis with discrete traits can be used [74], where transitions from one state to another are inferred along the phylogeny as a continuous time Markov chain model [75] (e.g. H5N1 in Asia [63] and H7N3 in North America [64]). Discrete trait analyses can be extended by parameterizing the transition rate matrix as a log–linear function of various potential covariates in a generalized linear modelling framework, to identify the host species or environmental factors associated with the observed spatial spread [7,76–78]. When the additional feature of interest is continuously distributed, e.g. location as latitude and longitude, Brownian random motion walks can be used to model the diffusion of the trait along the tree corresponding to the dispersal history of the pathogen [79,80]. The impact of environmental factors on virus dispersal can be estimated by correlating the distances along branches of the trees with the ‘resistances’ resulting from the diffusion path through landscapes of environmental variables using R package SERAPHIM [81].

In addition to trait-based approaches, BASTA [82] and MASCOT [83] make use of structural coalescent approximations [84,85] to reconstruct evolutionary trees while considering the size of the different sub-populations involved in the meta-population, improving inference of the migration rates between sub-populations. Finally, by combining epidemiological data and recent phylogenetic inference techniques, several methods such as SCOTTI [86], Outbreaker [87] and Beastlier [88] are now able to reconstruct, with some success, the transmission tree of only partially observed epidemics.

**Figure 4.**
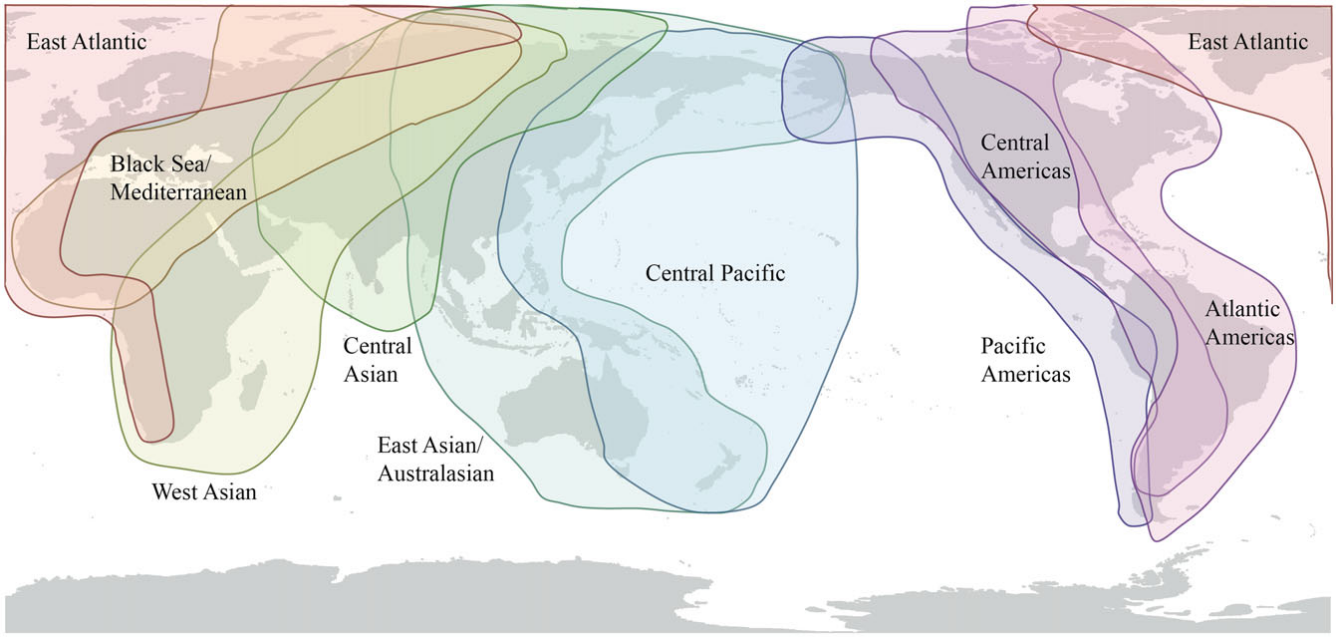
Flyways of migratory water fowl. Flyways run approximately north–south, and also overlap in northern regions, including in Siberia, Greenland, Alaska and across the Bering straits, which allows occasional transmission of influenza viruses between North America and Eurasia. Flyways from http://wpe.wetlands.org/Iwhatfly.

**Figure 5.**
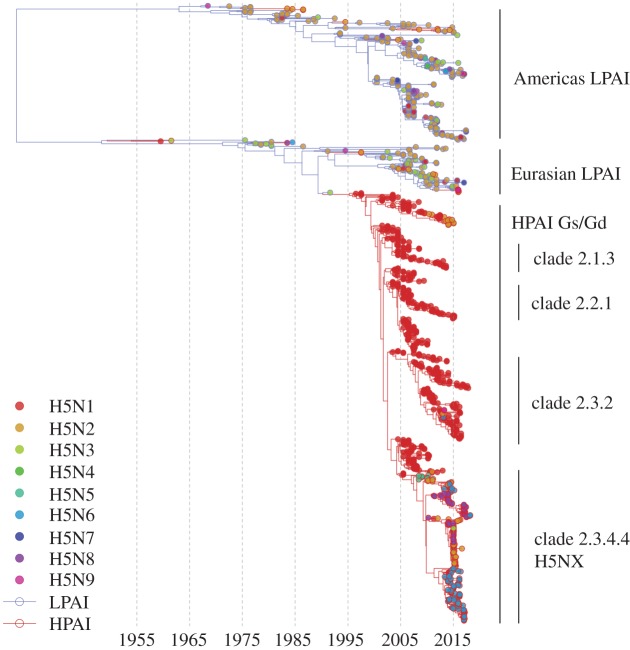
Time-scaled tree of a stratified subsample of H5 segment 4 (HA). The tree represents the known diversity of LPAI (blue branches) and HPAI (red branches). Tips are represented as circles and coloured by neuraminidase subtype from H5N1 (red) to H5N9 (magenta).

The effect that bird migratory flyways (figure 4) have on the global circulation of IAV can be seen in the phylogeny of all segments, where two very distinct major clades corresponding either to the Americas or to Asia, Europe, Africa and Australasia can be observed [65]. Estimates of the time to most recent common ancestor (TMRCA) of these clades varies by segment and method, but appears to be in the region of 100 years, a value close to the root of the major contemporary human, swine and avian lineages [40], and as such represents a deep divide (also evident in figure 5). Although viral dissemination via wild birds can be thought of as occurring along flyways, different species have different migration patterns, and these general flyways overlap, as indicated in figure 4. Consequently, cross-flyway (e.g. [89]), and intercontinental transmission of avian IAV by wild birds does occur. In a study of northern pintails, Koehler *et al.* found intercontinental transmission of LPAI connecting the East Asian-Australasian flyway to the Pacific Americas [92], while in 2014/2015 HPAI H5N8 was introduced into North America by a similar route [69,93]. There is also evidence for transfer of IAV genes from the Americas to Eurasia [94].

However, even if migratory birds might be good vectors, transmission patterns indicate that circulation is partially maintained through trade of infected domestic birds [91,95,96]. For example, the long-distance expansion of H5N1 HPAI viruses in 2004 was found to be caused by human-directed movements of domestic poultry [96], and both LPAI and HPAI virus circulation was found to be driven by human activities in China [76]. Therefore, it is clear that the worldwide spread of avian IAV results from synergy between trade of infected domestic birds and wild bird movement through migratory flyways [91,97].

New HPAI strains are thought to emerge from an LPAI progenitor (see box 1) following their introduction into domestic bird populations (e.g. [98,99]). Since domestic ducks can share the same habitat, water and food as wild waterfowl [100,101], their presence and concentration are thought to make them key intermediate hosts between wild birds and poultry, and consequently they play an important role in the emergence and circulation of HPAI strains, especially in Asia [101–103]. The bridging role of domestic ducks between wild birds and domestic Galliformes has been particularly emphasized in the H7N9 IAV outbreaks in China [104], most notably in areas where high concentrations of free-grazing ducks live in close contact with potentially infected wild birds, such as the Poyang and Dongting Lakes [100]. Agricultural practices, such as the release of high quantities of juvenile ducks in paddy fields prior to the arrival of the wild birds, might further exacerbate transmission and circulation of the virus between wild and domestic animals.

## 3. Rise of highly pathogenic avian influenza

As noted above (box 1), H5 and H7 avian strains of IAV are further classified as highly pathogenic on the basis of their ability to cause disease and mortality in chickens [25].

Over 20 years ago, phylogenetic analysis of HA sequences indicated that HPAI strains had independently evolved on separate occasions from ancestral LPAI viruses [16]. This has since been confirmed by many detailed sequencing analyses of outbreaks where direct LPAI precursors have been identified, even down to individual poultry sheds (e.g. [105,106]). Dhingra *et al.* performed a meta-analysis of H5 and H7 outbreaks from 1959 to 2015 and found 39 independent LPAI to HPAI transition events [107], and the majority of these (37 out of 39) were associated with commercial poultry farming. As HPAI in poultry has a rapid onset and high mortality rate, farm outbreaks can be short lived, partly because a large percentage of the birds die in a few days, but also because HPAI is a notifiable disease with mandatory control measures, including culling remaining birds and movement bans to limit the spread to neighbouring areas [25]. However, on some notable occasions HPAI outbreaks have caused major losses in domestic birds (see [13] for a review up to 2008). Apart from the widespread HPAI H5s originating in Asia from 1996 onwards, and the associated H7s which are described in detail next, other outbreaks resulting in huge impacts (i.e. the death or destruction of more than 1 million birds) include: Pennsylvania, USA 1983 (H5N2) [108], Mexico 1994 (H5N2) [109], Italy 1999 (H7N1) [110], The Netherlands 2003 (H7N7) [111] and British Columbia, Canada 2004 (H7N3) [112].

## 4. Highly pathogenic H5N1 viruses: 1996–2009

In 1996, an HPAI H5N1 virus was found in commercial geese in the Guandong Province, China (A/Goose/Guangdong/1/96), which was thought to originate from H5 viruses in wild migratory birds [113]. These Goose/Guangdong (Gs/Gd) lineage strains gave rise to outbreaks of HPAI H5N1 in chicken farms in Hong Kong in 1997 that further led to fatal human infections [46,114–118]. Surveillance of live bird markets revealed that H5N1 was widespread in poultry [119,120], and because of the zoonotic risk, all poultry in Hong Kong were culled in the winter of 1997/1998 [120]. This was partially successful in that the ‘HK-97’ lineage of HPAI H5N1 virus became extinct in Hong Kong [121]. However, reassorted Gs/Gd-like H5N1 viruses re-appeared in 2001 [95,121–123]. Phylogenetic studies of whole virus genomes revealed that around 1996–2002 several different genotypes of H5N1 arose from reassortment events between the original HPAI H5N1 virus with other LPAI strains circulating in both domestic and wild bird populations [95,121–123]. By 2003, one genotype (Z) had become dominant [123], and in addition to further human cases in Hong Kong in 2003, there were poultry outbreaks in mainland China and other countries in Southeast and East Asia [124]. Associated with these poultry outbreaks, there were also fatal human cases in Vietnam, Thailand and China [125].

In the spring of 2005, a mass die-off of wild birds occurred at Qinghai Lake in west China [126]. The majority of dead birds were bar-headed geese (*Anser indicus*), infected with a mix of two previously identified HPAI H5N1 genotypes (V and Z) [126]. The outbreak virus was thought most likely to have originated from poultry in southern China and had been transported by migratory birds to Qinghai Lake [95,126,127]. This episode was particularly concerning because it showed that the HPAI virus could be transmitted within wild migratory bird populations, with the consequent further possibility of spread to the south Asian subcontinent and/or to Europe [127]. Furthermore, the virus contained a mutation in a polymerase gene (PB2 627 K) that had been shown to increase H5N1 virulence in mice, a model for mammalian infection capability [128].

HPAI H5N1 spread out from the Southeast Asia region into Europe, the Mediterranean and Africa through the rest of 2005 and 2006 [129], with the first reports of infected birds in Russia and Kazakhstan in July 2005 and detections in Turkey, Romania and Croatia in October 2005. A single H5N1-infected migratory flamingo was found in Kuwait in November 2005 [124], and by February 2006 Iraq and Iran were reporting virus in backyard poultry and wild birds, as well as human and domestic cat cases [130]. In January and February 2006, there were several first detections reported in southern and western European countries [124]. H5N1 was first reported in Africa in Nigerian poultry in February 2006 [129], closely followed by reports of poultry outbreaks in Egypt [129,131–133]. The virus continued to spread in Africa, west and northwards in Europe and through the Middle East and South Asian subcontinent in 2006 and 2007.

Questions had already been raised about H5N1 as the source of the next influenza pandemic [134,135], so its spread out of Asia was of continued concern. Reinforcing these fears, by early 2009 H5N1 was endemic through southeastern Asia, had spread through Eurasia and Africa, and was established in domestic bird populations. It had also caused several hundred human deaths with estimated case-fatality ratios of around 30–80% depending on the country [125] (although the real case-fatality ratio could be different due to case definitions, survey methods and reporting [136]). There was also evidence for limited person to person spread [137,138].

Fortunately, there was no human transmissible H5N1 pandemic in 2009, something that might have caused the deaths of millions (assuming that a human transmissible form retained high pathogenicity). Instead the world was gripped by the much milder 2009 H1N1 ‘swine flu’ pandemic [44,139]. Despite mostly mild symptoms in humans, the lack of immunity to this quite different strain from the previously circulating H1N1 seasonal virus meant that a significant fraction of the population had probably been infected. Combining serology studies from 19 countries, an age-adjusted cumulative incidence estimate of just under a quarter (24%) of the population was obtained [140], and the excess mortality in the first year was estimated as between 151 and 575 thousand people [141]. The pandemic H1N1 strain went on to replace the previous H1N1 human seasonal IAV, and now co-circulates alongside seasonal H3N2 IAV and influenza B viruses in the human population.

## 5. Emergence of H7N9 in poultry and humans

Even as the world's attention was on HPAI H5N1 IAV and the new pandemic H1N1 virus in humans, transmission of LPAI wild bird viruses to domestic ducks, reassortment with co-circulating domestic viruses, and onwards transmission to poultry populations resulted in circulating lineages of H7N9 and H7N7 viruses [104,142]. The internal gene segments were from a combination of H9N2 virus lineages circulating in poultry [143–145], one of which probably also donated internal segments to the HPAI H5N1 viruses [104,143,144]. The H7N9 viruses proved to be zoonotic as the first human cases were found in February 2013 in Shanghai and Anhui, China [146,147]. From February 2013 to July 2018, there have been 1625 confirmed human cases and 623 deaths, mostly in China [148,149].

Between February 2013 and July 2017, there were five seasonal waves of human H7N9 infections [150], with waves 2–5 starting around October and lasting until around June. Up until the fifth wave, the H7N9 viruses found as part of surveillance in live bird markets were LPAI according to HA sequence, and asymptomatic/mild for chickens [151,152], but caused a range of symptoms in humans including severe respiratory distress and death [146,147]. However, there were mutations in the HA, including Q226L (using H3 HA numbering), that were associated with increased binding to human sialic-acid receptors and airborne transmission between mammals [142,153,154]. However, although these viruses were shown to be transmissible in ferret studies [155], human to human transmission actually remained limited [156] and most cases were associated with contact with infected poultry or live bird markets [157]. To control the disease, live poultry markets in affected central urban areas were closed [157], and the total number of human cases per wave decreased from wave 2 to wave 4 [148]. Nevertheless, the virus, asymptomatic or with mild clinical signs for birds, continued to circulate in poultry populations via trade in China, and diversified into several clades [158]. The fifth wave (starting September 2016) saw a rapid increase in the number of human cases and geographic expansion out of southern and eastern China despite the surveillance and control measures [159]. Also during the fifth wave, HPAI versions of H7N9 were detected in chickens and in human cases in December 2016 and January 2017 [148,160,161].

Phylogeographic studies indicated that the HA multi-basic cleavage site was estimated to have been inserted into an LPAI H7N9 lineage circulating in the Yangtze River Delta region around May 2016, which later went on to reassort with one of the other H7N9 clades in the Pearl Delta region and H9N2 [162]. As LPAI H7N9 can cause severe illness and death in people, it is unsurprising that HPAI also does, but it has been suggested that disease progression is more rapid for HPAI than LPAI infections [160]. Also of concern was the presence and ease of acquisition of E627 K or D701N mutations in PB2 in the human isolates, since, similarly to HPAI H5N1 cases, these are associated with increased virulence and adaptation in mammals [160,163].

A nationwide vaccination programme for poultry in China was begun in September 2017 by the Chinese Ministry of Agriculture [164]. Recombinant H5 and H7 bivalent inactivated vaccines were used [164] with subsequent testing of post-vaccination immunization, and also continued surveillance. The overall post-vaccination rate of immunization was in excess of 80% (the target was 70%) although there was a considerable variation between provinces. Only a few (11 out of over 80 000 in December 2017) samples from birds or their environment tested positive for H7N9, and there were only three human cases of H7N9 in the time period (September 2017–June 2018) expected to show a sixth wave of human infection, compared with over 700 the previous year. The risk of spread of H7N9 to surrounding countries in Southeast Asia is still considered to be moderate via live bird trade, but low for poultry products and negligible for onward spread via wild birds [165]. The risk of human occupational exposure in live bird markets is also considered to be moderate to low [165]. The small number of positive poultry samples found in the winter of 2017/2018 and near-absence of a sixth seasonal burst of zoonotic infections suggest that the policy of mass poultry vaccination has been successful in reducing the prevalence and risk of infection from H7N9 viruses.

## 6. Diversification of highly pathogenic H5 viruses: 2009–2018

Since its first detection in 1996, the HPAI H5N1 Gs/Gd virus lineage has undergone reassortment of internal protein-coding segments and diversification of the HPAI H5 gene into ten major clades [166,167]. In clade 2, there are several sub-lineages that are notable for the number of birds they have infected, their geographical spread, and spill-over to humans, including 2.1.3 (Indonesia), 2.2.1 (Egypt), 2.3.2 (Southeast Asia), 2.3.4 (widespread) [167]. Although the H5 HA is paired with an N1 NA for most outbreaks and continued circulation, sub-clade 2.3.4.4 is unusual for HPAI H5 Gs/Gd in that it has been undergoing frequent reassortment with LPAI strains since 2008. In the process, sub-clade 2.3.4.4 HAs have acquired N2, N5, N6 or N8 NAs, and these viruses are collectively known as H5NX [167], as indicated in figure 5.

H5NX viruses were detected in poultry farms and live bird markets, particularly in ducks, as part of the ongoing surveillance effort in China from 2009 onwards [168–171]. H5N5 viruses were initially more prevalent [168,172], while H5N8 viruses seem to have been circulating in the domestic duck population at low levels, as they were first detected in eastern China in 2010 [170] (A/duck/Jiangsu/k1203/2010(H5N8)) but then apparently disappeared. In 2013, H5N8 viruses re-appeared in eastern China in 2013 with some internal segment reassortments from H5N1 strains [173], were detected in wild mallards (e.g. A/mallard duck/Shanghai/SH-9/2013(H5N8)) [174], and found in apparently healthy ducks and geese in a live bird market in Guangdong, southern China in 2013–2014 [175].

In January 2014, outbreaks of H5N8 were reported in South Korea [176]. These viruses had high similarity to A/duck/Jiangsu/k1203/2010 (H5N8) for the HA and NA segments, but had internal segments from at least two different lineages co-circulating in eastern China [176,177]. From detailed time-scaled phylogeographic analysis, it was inferred that H5N8 had entered South Korea via overwintering wild waterfowl which subsequently infected domestic ducks [70,177].

### (a) Global transmission of H5N8 on migratory flyways

The following autumn and winter of 2014/2015 saw widespread H5N8 detections in wild birds and multiple outbreaks in domestic flocks in Japan, Europe and North America [69,178]. Both phylogeographic analysis of sampled sequences and knowledge of bird migration patterns indicated that the virus was transported by migrating wild Anseriformes from the eastern Asia region, up to the summer breeding grounds in the north by the East Asian flyway, and then down into Europe via the East Atlantic flyway or to North America via Beringia (Pacific and Central flyways) [69,93,179]. This latter event was notable as the first recorded occasion, nearly 20 years after first isolation, in which a virus bearing an HPAI H5 Gs/Gd lineage HA had crossed the Bering Straits.

In North America, the H5N8 virus was initially detected in wild birds in Washington State, USA and British Columbia, Canada where it reassorted with North American LPAIs, acquiring local internal protein-coding segments and N1 or N2 neuraminidases [93,178–180]. The new reassortant H5N2 virus (Asian H5, North American N2) rapidly spread through USA commercial poultry flocks from January to June 2015 [181]. Around 50 million birds were infected and/or destroyed as part of control measures, and with a cost of at least 1 billion US$ to the industry, this is the most expensive recorded North American avian influenza epidemic to date [182,183]. The control measures were successful, however, and by the autumn of 2015, H5N2 was not reported either from industry [182], nor from North American wild bird surveillance studies [184].

Clade 2.3.4.4 H5 viruses also receded from Europe in the spring of 2015, having caused outbreaks in wild birds and domestic flocks in several countries including Germany, The Netherlands, the UK, Sweden, Italy and Hungary [69,177,185–190], and were not detected in the following winter season (2015/2016). In southwest France from late 2015 to summer 2016, infection with HPAI H5 viruses of H5N1, H5N2 and H5N9 subtypes were reported from waterfowl, chicken and guinea fowl farms [191], but these viruses were reassortants descended from LPAI circulation in the Eurasian virus pool which had evolved a multi-basic cleavage site de novo [192], and were not related to HPAI H5 clade 2.3.4.4.

### (b) H5N6: a new threat in Asia

In Asia from 2014 onwards H5NX lineages continued to circulate in wild and domestic bird populations, and as well as H5N8, two different H5N6 reassortant lineages were detected in Sichuan and Jiangxi provinces of China, respectively in 2014 [193–195]. Both of these lineages spread within poultry in China [194,196,197], and to wild bird populations [198]. One of the lineages, having acquired internal protein-coding segments from H5N1/H7N9 lineage viruses, and a neuraminidase N6 with a stalk deletion (a poultry adaption), has also caused sporadic human infections [163,199–201]. Clade 2.3.4.4 viruses generally, and especially H5N6, seem to be successful in poultry populations in China and have spread to other Southeast Asian countries including Vietnam, Laos and Korea [196,202–205]. It is also likely that H5N6 has been transmitted on the East Asian–Australian flyway by wild migratory birds [206], since Japan has been bombarded with H5N6 reassortants [207,208], and it is also possible that H5N6 was introduced into The Philippines via wild birds for the first time in the summer of 2017 [209].

### (c) Repeat invasion of Europe by H5NX

The autumn/winter of 2016/2017 saw the return of H5N8 into Europe from Asia [210] by a sister clade of the 2014/2015 viruses (rather than a direct continuation of the 2014/2015 lineage). The pathogenicity of the new 2016/2017 H5N8 viruses appears to be greater in ducks and has caused more deaths in wild birds [211] than the previous 2014/2015 H5N8 viruses [212]. Additionally, the transmission of the 2016/2017 viruses out of eastern Asia was by a more southerly route than previously observed, with detection in May 2016 around Qinghai Lake in wild birds [213], near Uvs-Nuur Lake, near the Russian–Mongolian border, in June 2016 [210], and finally in India, the Middle East and Europe in November 2016 [211,214–218] as a result of winter southward migrations. Reassortment events between the incoming HPAI H5N8 strain and other co-circulating LPAI strains occurred frequently in the 2016/2017 season, including the generation and transmission of H5N5 viruses. Multiple incursions of these HPAI H5NX strains into European countries occurred [217], causing the worst epidemic so far in Germany, affecting both domestic and wild birds [214]. In the following season (2017/2018), a new reassortant H5N6 derived from the 2016/2017 H5N8 viruses (i.e. different from the H5N6 that infected humans in Asia) was detected in the UK, The Netherlands and Germany in December 2017 and early 2018 [219,220]. Most recently (September 2018), The Netherlands and Germany have reported their first H5N6 detection of the autumn 2018 season [221,222].

## 7. Concluding remarks

In this brief history of bird flu, we have seen that current avian influenza virus strains have been circulating and diversifying in wild bird populations for at least the last 100 years. Wild migratory birds can transport IAV along their migration routes, and contact between wild and domestic avian populations sometimes results in transmission between the two. Direct transmission of the virus from wild birds to humans appears to be very rare (or non-existent), presumably due to the low frequency of contact between the two populations; however, transmission from domestic avian species to humans does occur, especially in live bird markets in Asia. It is clear that H5 and H7 viruses have the capacity to evolve (on multiple occasions) an HPAI phenotype, probably as result of transmission in high bird density settings and the susceptibility of chicken and other domestic Galliformes species. In recent years, one such H5 lineage has become widely established in Asian domestic bird populations. Both H5 and H7 HPAI viruses have been sporadically transmitted to humans from domestic poultry, and (for H5 at least) been transmitted back into wild populations. However, because HPAI does not necessarily kill its anseriform hosts, reassortment with co-circulating LPAI viruses can occur, furthering evolution of the virus, while the low severity symptoms allow the long-range and intercontinental transport of the disease.

In some senses, the dynamics of human influenza in humans and avian influenza in birds are similar—both can be thought of as stratified into layers with different connectivity: age for humans—with locally moving children and long-range moving adults; and domestic and wild species for birds—with domestic birds moving via trade and Anseriformes by long-range migration. However, unlike human IAV, where reassortment between the few dominant subtypes is rare, reassortment is a common feature for avian IAVs, especially in wild bird populations. Consequently, avian IAVs are far more diverse and more easily generate novel strains than the more specialized human viruses.

Looking to the future, we should expect the emergence of more HPAI strains. Experience teaches that this has previously occurred somewhere in the world approximately once or twice per decade; and the fundamental driver of leaving H5 and H7 LPAI viruses uncontrolled in a host-dense environment until de novo mutation into HPAI forms occurs has not been removed. Also, it seems quite possible that HPAI H5 will continue to circulate and diversify, especially for clade 2.3.4.4 because it does not necessarily cause severe clinical signs in its wild hosts and is therefore capable of silent spread. Hence increasing biosecurity and vaccination in domestic poultry are likely to be important strategies to keep outbreaks in these populations to a minimum. Ongoing avian influenza virus spill-overs into human cases suggest that zoonotic bird flu is a continued threat to human health; however, the apparent success of the H7N9 vaccination programme in China suggests that it is possible to control virus circulation in domestic birds and thus vastly reduce the number of human infections and the risk of ongoing human to human spread. Therefore, if we continue the disease surveillance programmes in avian, human and other domestic animal populations, and control avian influenza in domestic avian populations, then we can surely reduce the risks of a new human avian influenza pandemic.

## Supplementary Material

Supplementary Text

## Supplementary Material

Data for segments 1,2,3,5,7,8 - 10279 taxa

## Supplementary Material

Data for segments 1,2,3,5,7,8 - 8809 taxa for display

## Supplementary Material

Data for segment 4 subtype H5 - 1177 taxa

## Data accessibility

Nucleotide sequence data, phylogenetic trees and associated information tables for the global subsample of internal segments, and the set of subtype H5 segment 4s can be found in the electronic supplementary material and are available from the Dryad Digital Repository: https://doi.org/10.5061/dryad.5nb504n [223]. The phylogenetic trees may also be explored with an online tool; please see the supplementary text for details.
