# Supplementary material for: A brief history of bird flu
Source: Philos Trans R Soc Lond B Biol Sci. 2019 May 6;374(1775):20180257. doi: 10.1098/rstb.2018.0257 (PMC6553608; doi:10.1098/rstb.2018.0257)
Supplement: Supplementary Text [file rstb20180257supp1.pdf]

Supplementary Text

Sequence Data and Stratified Subsampling Procedure

40249 Influenza A whole nucleotide genomes sets (325603 sequences) were downloaded from the NCBI Influenza Virus Resource on 27th July 2018 [1], for any host, any country, any subtype and any date, with fasta definition line set to the following:

>{serotype}_{host}_{accession}_{strain}_{country}_{year}/{mont}/{day}_{segname}

Using a custom R-shiny subsampling tool ("FluSubsampling"), 38359 unique whole genome sets were detected, and from these 2 sequences per strata were selected. The strata were Year, Country or State, Subtype and Host (see below), and resulted in 6901 unique categories, from which 10356 sequences were selected.

**Strata**	**Categories**
Year	82 unique years, 1902 to 2018
Country or State (if China, USA or Russia)	455 unique locations
Subtype (excluding mixed)	122 unique subtypes
Host<br>• only 'major' host species: Avian, Swine, Human, Equine, Canine (5 types)<br>• The Avian hosts were futher stratified into Bird Order (anseriformes etc)<br>• 4 bat genomes were added in later as outgroups	22 types (and bats added later)

Nucleotide sequences from each internal protein coding segment of the 10356 genomes (segments 1,2,3,5,7,8) were first trimmed to the start codons, and adjusted to be in frame, before aligning in 10 random blocks by MUSCLE in MEGA 7 [2]. One file per segment was reformed with manual adjustments, poor quality sequences were excluded (e.g. non-full length sequences, or sequences with excessive ambiguities), and the 4 bat sequences were added and aligned to the other sequences. This resulted in a selection of 10279 isolates with all internal genes present.

In order to display the large trees in ITOL (https://itol.embl.de/) [3] without node collapsing, only 10000 taxa may be used. Therefore we further subsampled the sequences to reduce categories which looked to be over-represented (due to field sampling effort) - Human sequences from the USA were further sampled to only 1 per year, per place, per subtype, and Avian sequences with hosts classified as Wild-ans-short (short range migrant anseriformes, e.g. mallards) were also subsampled to 1 per year per place. This resulted in a final data set for display of 8809 isolates with all internal genes present.

Segment 4 sequences were also extracted from the original 10356 genome set, and sequences of H5 subtype only were also trimmed to the coding regions and aligned using MUSCLE in MEGA 7 followed by small manual adjustments, and removal of low quality sequences. This resulted in a dataset of 1177 HA-H5 sequences.

Phylogeny Creation

Trees from Internal Segments

Neighbour joining trees from segments 1,2,3,5,7,8 were created using R package ape [4] with the genetic distances defined by a Tamura-Nei model, with a gamma distributed site to site variation (parameter = 1) and pairwise deletion between sites. The trees were rooted on the ancestor of the 4 bat sequences.

HA H5 BEAST MCC Tree

A time-scaled phylogeny was created from the HA H5 sequences dataset using BEAST [5] with the Tamura-Nei (TN93) nucleotide substitution model, uncorrelated relaxed log-normal clock model and constant population size tree prior. 4 independent MCMC chains were used, each consisting of at least 500000000 steps and sampling every 100000 steps. From these a posterior sample of 1000 trees (after 10% burnin was removed) was summarised in TreeAnnotator to give the maximum clade credibility tree (MCC tree).

Phylogenies Online

The final trees have been shared on ITOL under shared project https://itol.embl.de/shared/slycett
Links to individual trees are:

Internal Segments
Segment 1 https://itol.embl.de/tree/1292154611176571542817686
Segment 2 https://itol.embl.de/tree/1292154611193981542818024
Segment 3 https://itol.embl.de/tree/1292154611219351542818364
Segment 5 https://itol.embl.de/tree/1292154611261541542818902
Segment 7 https://itol.embl.de/tree/1292154611298851542819236
Segment 8 https://itol.embl.de/tree/1292154611344271542819666

HA (H5)
Segment 4 https://itol.embl.de/tree/1292154611241571536684286

Supplementary References

1. Bao Y, Bolotov P, Dernovoy D, Kiryutin B, Zaslavsky L, Tatusova T, Ostell J, Lipman D. 2008 The influenza virus resource at the National Center for Biotechnology Information. *J. Virol.* **82**, 596–601. (doi:10.1128/JVI.02005-07)

2. Kumar S, Stecher G, Tamura K. 2016 MEGA7: Molecular Evolutionary Genetics Analysis Version 7.0 for Bigger Datasets. *Mol. Biol. Evol.* **33**, 1870–1874. (doi:10.1093/molbev/msw054)

3. Letunic I, Bork P. 2016 Interactive tree of life (iTOL) v3: an online tool for the display and annotation of phylogenetic and other trees. *Nucleic Acids Res.* **44**, W242–W245. (doi:10.1093/nar/gkw290)

4. Paradis E *et al.* 2015 Package ‘ape’. *Anal. phylogenetics Evol. version* (doi:10.1109/TMECH.2007.897281)

5. Drummond AJ, Suchard MA, Xie D, Rambaut A. 2012 Bayesian phylogenetics with BEAUti and the BEAST 1.7. *Mol. Biol. Evol.* **29**, 1969–73. (doi:10.1093/molbev/mss075)
