# Supplementary figures and images for: A brief history of bird flu
Source: Philos Trans R Soc Lond B Biol Sci. 2019 May 6;374(1775):20180257. doi: 10.1098/rstb.2018.0257 (PMC6553608; doi:10.1098/rstb.2018.0257)

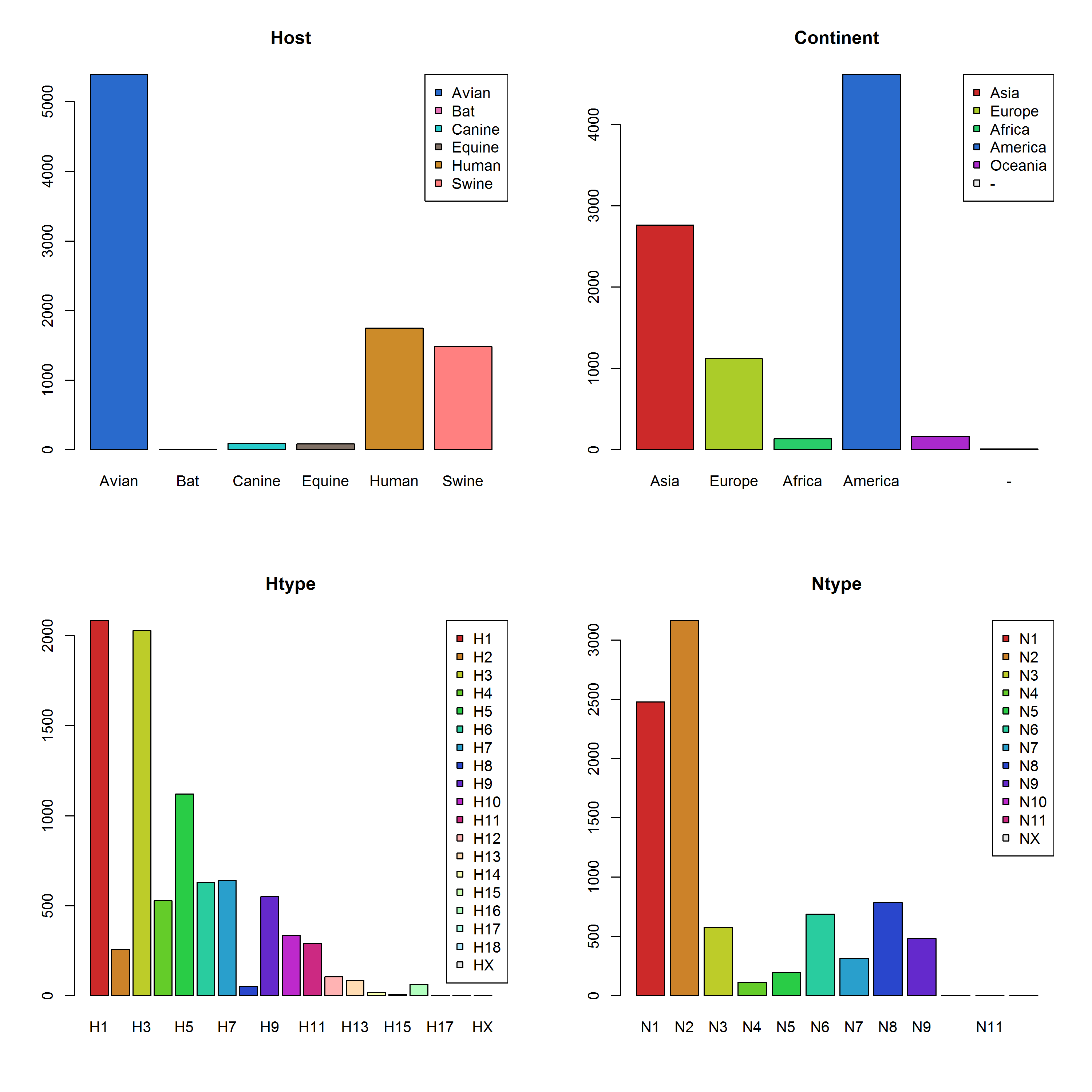

Supplement: Data for segments 1,2,3,5,7,8 - 8809 taxa for display [file rstb20180257supp3.zip › internals_subsample_for_display/hxnx_all_segs_8809_barplots.png]
